# Conditioned medium and secretome from epididymal epithelial cell cultures improve sperm kinetics and capacitation

**DOI:** 10.14202/vetworld.2023.1325-1332

**Published:** 2023-06-13

**Authors:** Luluk Yunaini, Dwi Ari Pujianto

**Affiliations:** 1 Doctoral Program for Biomedical Sciences, Faculty of Medicine, Universitas Indonesia, Jakarta 10430, Indonesia; 2 Department of Medical Biology, Faculty of Medicine, Universitas Indonesia, Jakarta 10430, Indonesia

**Keywords:** conditioned medium, sperm kinetics, secretome, phosphorylation

## Abstract

**Background and Aim:**

Sperm maturation occurs in the epididymis through interactions with existing molecules inside the lumen. However, the mechanism of epididymis molecular transfer is currently unclear. This study was aimed to determine the necessity of the epididymal epithelial cells (EECs) in the process of sperm maturation in terms of sperm kinetics and tyrosine phosphorylation.

**Materials and Methods:**

A true experimental research design was used in this study. The medium tested was a primary culture of mice caput epididymal cells (cells and culture medium), conditioned medium (CM) (supernatant of EECs), and secretome (CM filtered at 0.22 μm). Sperm was cocultured in EEC culture, CM, and secretome for 1, 2, 3, or 4 h. The original culture medium was used as the control. Sperm kinetic analysis was performed after the indicated times using computer-assisted sperm analysis, and tyrosine phosphorylation was detected using the Western blot technique.

**Results:**

A primary culture of caput EECs was successfully generated. The results showed increased sperm motility and progressive movement after 3 h of incubation (p < 0.05). There was a significant decrease in the average path velocity (VAP) values after 4 h of incubation (p < 0.05), but there was no significant change in the 1, 2, and 3 h incubation groups. The EEC culture-CM and secretome groups showed a significant increased progressivity and VAP percentage values compared with the control medium (p < 0.05). In terms of percentage motility, the culture and CM groups were significantly different from the control medium, but the secretome group was not.

**Conclusion:**

The sperm kinetics of sperm cultured in CM, secretome, and EEC were significantly increased after 3 h of incubation, suggesting that CM and secretome can be used to replace EECs, especially when analyzing molecules secreted by the epididymal epithelium during sperm maturation. The results of this study highlight the potential of CM and secretome as therapy mediums for sperm kinetic abnormalities.

## Introduction

The winding male reproductive tract known as the epididymis is the link between the testes and the vas deferens and functions as a place for the transport, maturation, and storage of sperm [1–3]. This organ comprises three segments: the caput, carpus, and cauda [2]. Each epididymis segment has a specific gene expression pattern that leads to region-specific protein secretion [2, 4–9]. The previous studies [7, 10, 11] have reported that the epididymal genes and proteins that play a role in sperm maturation are regulated by androgens and testicular factors. These genes are expressed from puberty to adulthood and mostly produce secretory proteins. Sperm maturation includes a progressive increase in motility and changes in movement patterns, such as circular motions in mouse sperm or vibrational movements in human sperm [12]. During maturation, the flagellum of the sperm becomes stiff and the neck area lacks flexibility to allow the movement of the sperm to be more rigid. In addition, the transit of the cytoplasmic droplets from the base of the sperm head at the end of the flagellum midpiece [13]. The activation of sperm motility is mediated by increased cyclic adenosine monophosphate and calcium levels and changes in the phosphorylation status of specific proteins [14–16]. The process of early sperm maturation in the epididymis occurs in the proximal part in terms of motility initiation. The process of further maturation occurs in the corpus, while the cauda acts as a storage area for sperm before ejaculation. [17].

When the sperm transits in the epididymis, the chemical composition of both the cell surface and the sperm cell interior undergo midifications. These modifications occur due to the addition and release of proteins, lipids, and sugars when sperm cells are in the lumen of the epididymis [9, 18–20]. Proteins obtained during maturation come from proteins secreted by epididymal epithelial cells (EECs), because sperm are silent cells for gene expression (transcription and translation) [21]. However, the mechanisms of epididymal protein transfer to the sperm are not fully understood. Three hypotheses have been proposed to explain the transfer process: (1) Absorption of soluble proteins secreted by the epithelium into the epididymal fluid [22]; (2) transfer of exosomes released from the epithelium of the epididymis; these exosomes contain epididymosome-containing protein, non-coding RNA, and lipid, which are transferred to the sperm while they pass through different regions of epididymis [23–25]; and (3) molecules are transferred directly from the epididymis to the sperm through the apical surface of the epididymal epithelium [26].

Primary cultures of epididymal cells have been successfully obtained in various species [27–29]. The interactions between the sperm and secretory proteins from the epididymis play an important role in the sperm maturation process. In this study, we carried out coculture of sperm using a variety of media in the form of EEC culture, conditioned medium (CM), and secretomes medium (SM) to provide an overview of the direct and indirect roles of EEC in the process of epididymal molecule transfer and to elucidate the mechanism of epididymal protein transfer.

This study analyzed the impact of sperm coculture medium (EEC, CM, and SM) on sperm kinetics and tyrosine phosphorylation. Time incubation plays a role in the kinetic effect of sperm; therefore, we also analyzed the optimal time of incubation for sperm coculture. We used a primary culture of mouse caput epididymis cells to determine the effect on sperm maturation in terms of sperm kinetics at the cellular level and tyrosine phosphorylation as an indicator of capacitation.

## Materials and Methods

### Ethical approval

This study was approved by the Medical Research Ethics Committee of the Faculty of Medicine, Universitas Indonesia (protocol no. 22-06-0738).

### Study period and location

The study was conducted from June to November 2022 at Animal Research Facilities (ARF) of the Indonesian Medical Education and Research Institute, Faculty of Medicine, Universitas Indonesia (IMERI FKUI) and Laboratory of Molecular Biology, Department of Medical Biology Faculty of Medicine Universitas Indonesia.

### Isolation and culture of caput EECs

Tissue samples were obtained from ten sexually mature, male ddY mice (8 weeks old) that were in good health. The epididymal tissue was stored in a 2-mL tube containing the transport medium (Dulbecco’s Modified Eagle Medium/Nutrient Mixture F-12 [DMEM/F12]) medium added with 100 units/mL penicillin and 100 mg/mL streptomycin). The caput epididymis was immediately processed within 30 min in the culture laboratory. The caput epididymal tissue was transferred to iodine solution for a few seconds, followed by washing with phosphate buffered saline (PBS) 3 times until the tissue was clean. The caput epididymis was dissected under sterile conditions in a Petri dish containing DMEM/F12 medium to remove connective tissue and fat. The caput epididymides were transferred to tubes containing 10 mg/mL collagenase in DMEM/F12 medium and incubated upright at 37°C for 30 min. The supernatant was discarded. The pellet containing epididymal tissue was transferred to DMEM/F12 medium containing 0.5 mg/mL hyaluronidase and then incubated at 25°C for 15 min. The supernatant was discarded, and the pellet was dissected under a dissecting microscope and added with 1% hyaluronidase solution, then incubated at 25°C for 30 min. The process of breaking down the tissue into cells was improved by repeated pipetting. The lysate was filtered through 80-mm copper mesh. The concentration of cells was calculated, and then, cells were planted on a 12-well cell culture plate to minimize the fibroblast contamination in the primary culture. Approximately 0.1 mg/mL of collagenase was added to the culture medium on the 1^st^ day. The culture medium was changed until the cells reached confluence.

### Characterization of caput epididymis primary cultured cells

The characterization of caput epididymis primary cultured cells was performed using the reverse transcriptase-polymerase chain reaction (RT-PCR) technique on the defensin beta 20 (*Defb20*) and aquaporin-9 (*AQP9*) genes. The *Defb20* gene was expressed specifically in the caput epididymis and *AQP9* was used for detecting principal epididymal cells. The research steps were total RNA isolation, cDNA synthesis, and RT-PCR. Total RNA isolation from primary cultured cells was performed using a Quick-RNA™ MiniPrep Plus Kit (Zymo Research, California, USA). The synthesis of cDNA was carried out using ReverTra Ace® qPCR RT Master Mix with a gDNA remover kit (TOYOBO, Tsuruga, Japan). The RT-PCR technique was used to apply the cDNA regions of the *Defb20* and *AQP9* genes and showed the expression of these two genes. Amplification was performed using two pairs of specific primers. The primer was designed using perlprimer3 software (https://primer3.ut.ee/). The sequence of primers used is shown in Table-1.

**Table-1:** List of primer sequences used in this study.

No.	Gene	Sequences	PCR product
1.	Defb20	Forward: 5’- TCGGGAGGATCTGAAGAC -3’, start 42 Reverse: 5’- GTTCTCCAGCTCATTAACAC -3’, start 236	194 bp
2.	Defb20	Forward: 5’- TCGGGAGGATCTGAAGAC-3’, start 42 Reverse: 5’- AGGAAAGAGACAGGACAG-3’, start 431	389 bp
3.	Aquaporin-9	Forward: 5’-GCATTTACTATGACGGACTC-3, start 570 Reverse: 5’- TCCACCAGAAGTTATTTCCA-3’, start 941	371 bp

Defb20=Defensin beta 20, PCR=Polymerase chain reaction

cDNA samples were amplified for 35 cycles with a pre-denaturation temperature of 94°C for 5 min, then entered into a cycle consisting of denaturation at 95°C for 30 s, annealing at 51°C for 30 s, and elongation at 7°C for 30 s. At the end of the cycle, the extension time was extended to 72°C for 7 min. The PCR product was stored at 4°C before electrophoresis in 1.6% agarose gel containing 0.1% ethidium bromide. Electrophoresis was performed at 90 V for 60 min. The visualization of the electrophoresis results was performed using an ultraviolet (UV) illuminator on a UV LONGLIFETM Filter (Spectroline, Merck, Jakarta, Indonesia) and photographed with a camera.

### Isolation of CM and secretomes from EEC cultures

When the EEC culture reached 90%–100% confluence, it was washed 3 times and culture medium was added. Then, the culture was incubated at 37°C in a humidified 5% (v/v) CO_2_ incubator. On a determined day, the supernatant was collected from the cell culture and labeled as “CM.” Secretome medium was obtained by filtering the CM using a 0.22-mm filter (Millipore, Bedford, MA).

### Sperm-EEC coculture

Sperm was cocultured in EEC culture, CM, SM, and original medium culture (MC; control) as a medium control. Sperm was incubated with various media at 37°C for 1, 2, 3, and 4 h.

### Sperm kinetics analysis using computer-assisted sperm analysis (CASA)

Specimens were collected for sperm kinetics testing using a disposable 4-cell Leja 20-mm CE chamber (Leja Products, Nieuw-Vennep, Netherlands) combined with (CASA; Hamilton Thorne, IVOS II, USA). Computer-assisted sperm analysis is a computer system with a high-resolution camera connected to a phase-contrast microscope. Analysis for one field of view takes only 1 s. The sperm kinetic parameters measured in this study were the percentage of motility, the percentage of progressive sperm, and the average velocity of sperm in their movement path (average path velocity, average pathway velocity [VAP], μm/s).

### Detection of tyrosine phosphorylation using Western immunoblotting

Western immunoblotting analysis was performed to examine tyrosine phosphorylation in the sperm after incubation in coculture medium. Twenty micrograms of sperm protein were separated on 10% sodium dodecyl-sulfate polyacrylamide gel electrophoresis and then transferred to a 0.45-mm hybond polyvinylidene difluoride (PVDF) membrane (GE Healthcare, Germany). The membranes were blocked with 3% bovine serum albumin (BSA) for 1 h at 25°C. The membrane was subsequently incubated with the primary antibody (anti-phosphotyrosine antibody; Santa Cruz, USA; 1:500) overnight at 4°C. The membrane was washed with 1× tris-buffered saline with 0.1% Tween® 20 detergent (TBST) for 5 min 3 times and then incubated with a secondary antibody (goat anti-mouse immunoglobin G-horseradish peroxidase (IgG HRP) conjugated; Santa Cruz, USA; 1:1000) for 2 h at room temperature. The membrane was washed 3 times with 1× TBST for 5 min and visualized using an ECL plus Western blot detection system (Thermo Fisher Scientific Inc., Hampton, USA). The antigen-antibody reaction was detected using a Luminescent Image Analyzer (Imagequant LAS 4000, Sweden).

### Statistical analysis

Data analysis was conducted using the Statistical Package for the Social Sciences version 25 (IBM SPSS Statistic, Chicago, USA). The data obtained were analyzed by univariate, bivariate, and multivariate analysis. A bivariate test was conducted to test the parameters between the independent and dependent variables. Analysis with more than one independent variable was analyzed using a multivariate test. The data with p < 0.05 were considered significant.

## Results

### Characteristics of mouse caput epididymal primary cell culture

**Figure-1:**
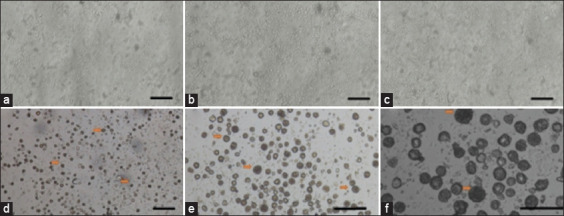
Primary cell culture from the epididymal caput tissues in DMEM/F2. (a-c) Culture cells derived from caput epididymis tissue showing homogenous morphology after culture for 1, 3, and 7 days, respectively. (d-f) Population culture cells showing more mature, heterogeneous size, and morphology after the second passage (with magnification 100×, 200×, and 400×, respectively) Arrow: mature cell and proacrosomal vesicles. Bars 100 μm.

Epididymal cells were isolated from the epididymis of 8-week-old mice. On day 0, the cells were clumped, but from day 1, single cells appeared to stick to and coat the plate (Figure-1a). The cells grew from the edge of the plate toward the center. On day 3 (Figure-1b), the cell-covered area on the plate was increased. On day 7 (Figure-1c), the cell-covered area was decreased compared to the 3^rd^ day but was higher than the 1^st^ day. Cells experienced another increase in volume starting on day 10, which continued until day 21. All cells showed irregular shapes (Figures-1d–f); this morphology is characteristic of the aging process that ends in death.

**Figure-2:**
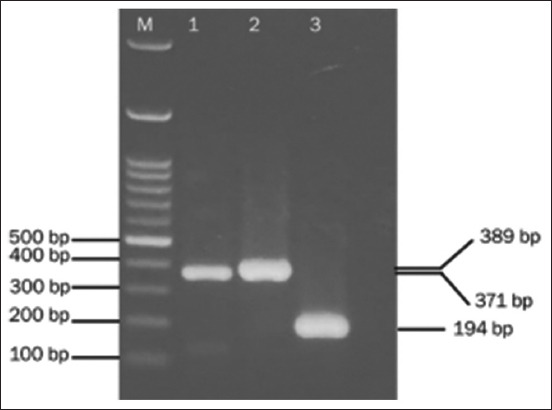
Electrophoretic description of the results of amplification using reverse transcriptase-polymerase chain reaction technique for the expression of Defensin beta 20 (wells 2 and 3) and Aquaporin3 (well 1) as markers for characterization of mouse epididymis caput cells. M: DNA ladder 100 bp.

After obtaining the primary culture of the caput cell epididymis, we confirmed cell specificity by checking the expression of two genes (*Defb20* and *AQP9*) that are specifically expressed in the caput epididymis. Genes were detected by RT-PCR. In the analysis of *Defb20* expression, two pairs of primers with different sizes were used (the entire mRNA and a portion of the RNA with inter-exon positions). Aquaporin-9 is a marker for principal epithelial cells in the epididymides of mice. Characterization using the RT-PCR technique on the *Defb20* and *AQP9* genes showed positive results, where bands matched the size of the target gene in the electrophoresis results (Figure-2). This result showed that the culture of cells grown and developed on the plate was mouse epididymis caput cells.

### Optimization of incubation time and the response of a medium type of sperm coculture to sperm kinetics

**Figure-3:**
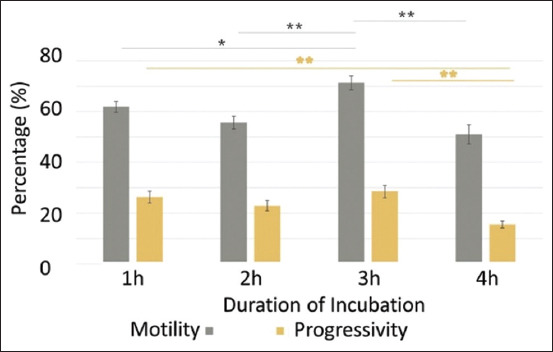
The graph between the percentage of sperm motility and progress against incubation time (1–4 h). The analysis used was a multivariate analysis of variance test with a *post hoc* test for motility using the Games-Howell test while progressive using the Bonferroni test. Mean ± SE; *p < 0.05, **p < 0.01. SE=Standard error.

Our results showed that sperm cells that had been incubated for 3 h had the highest percentage of motility, and the progressiveness was significant compared with incubation for 1, 2, and 4 h (Figure-3). In the analysis of the progressive movement of sperm, sperm that had been incubated for 1 h showed significant differences from sperm that had been incubated for 3 or 4 h. Cells that had been incubated for 4 h showed a progressive decrease in movements compared to sperm that had been incubated for 1 or 3 h, with the highest percentage at 3 h of incubation at 28.5%.

**Figure-4:**
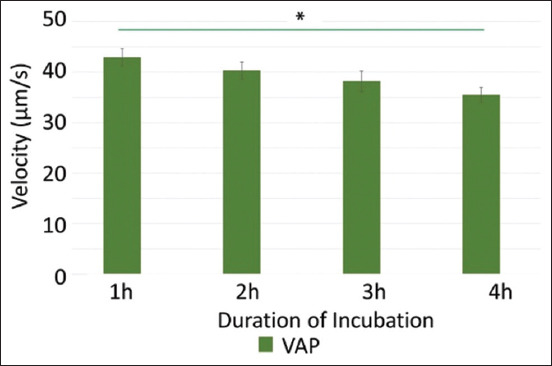
Graph of sperm velocity (VAP) and incubation time (1–4 h). Using the *post hoc* test, the Bonferroni test showed a significant difference between the 1 h and 4 h incubation groups with *p < 0.05. Mean ± SE. SE=Standard error, VAP=Average pathway velocity.

In addition to the analysis of the percentage of motility and progress, an analysis of the VAP value was also carried out. The results showed that sperm with a 1 h incubation time was significantly different from that with a 4 h incubation time (Figure-4), where 4 h incubation decreased the VAP value when compared to the 1, 2, and 3 h incubation times. The highest VAP value (42.9 μm/s) was found in sperm that had undergone 1 h of incubation. The results showed that the optimal incubation time in sperm coculture was 3 h, with the highest motility and progressive percentage values and VAP values that were not significantly different from the group with the highest VAP values.

**Figure-5:**
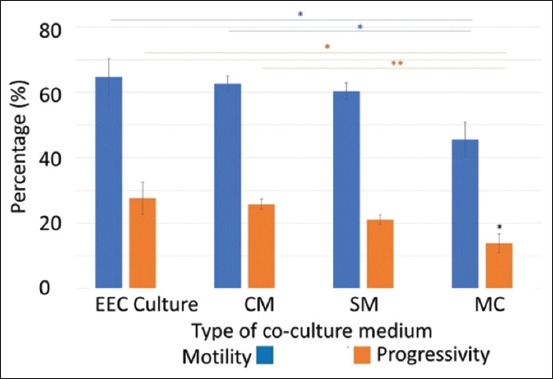
Graphic of the percentage of motility and progressive movement of sperm in various coculture mediums. A multivariate analysis of variance test was carried out with a Bonferroni *post hoc* for motility and the Games-Howell test for progressive with a significance value of *p < 0.05, **p < 0.01. Mean ± SE. EEC=Epididymal epithelial cell, CM=Conditioned medium, SM=Secretome medium, MC=Medium control, and SE=Standard error.

**Figure-6:**
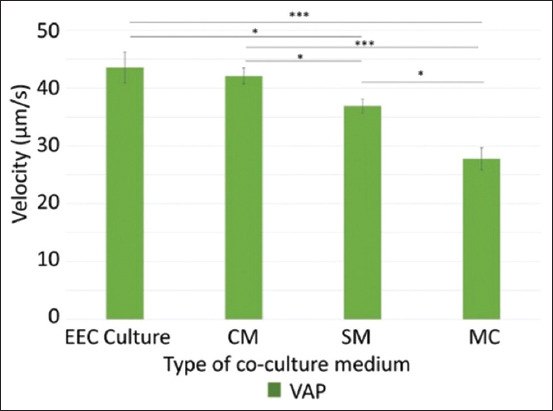
Graphic of sperm velocity values (VAP) for various types of sperm coculture medium used. The statistical test used one-way analysis of variance analysis with Bonferroni’s *post hoc* test with a significance value of *p < 0.05, ***p < 0.001. Mean ± SE. EEC=Epididymal epithelial cell, CM=Conditioned medium, SM=Secretome medium, MC=Medium control, and SE=Standard error, VAP=Average pathway velocity.

In addition to analyzing the optimal incubation time, an analysis was also carried out on the effect of the type of coculture media used on sperm kinetics (motility percentage, progressive movement percentage, and VAP). Variations of coculture media were used (EEC culture, CM, SM, and MC). The results showed that there was a significant increase in the percentage of sperm motility in the EEC culture and CM groups when compared to the MC group, but no significant difference when compared to the SC group (Figure-5). The highest percentage of motility was in the EEC culture (64.7%), while the lowest was in the MC (45.6%). Progressive movement of sperm showed a significant increase in EEC culture and CM compared with MC. The relationship between VAP values and the media used is shown in Figure-6. The results showed that there was a significant increase in VAP values for sperm cells in the EEC culture, CM, and SC medium compared with MC. The VAP values in the cell, CM, and SM groups were 43.5 μm/s, 42.1 μm/s, and 36.9 μm/s (p < 0.05), while the VAP value in the control medium group was 27.8 μm/s.

**Figure-7:**
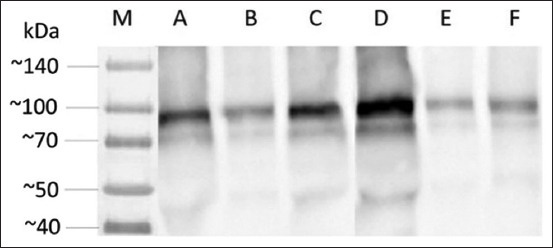
Western blot results for the analysis of tyrosine-phosphorylated protein on a variety of coculture mediums. (a) EEC culture with an incubation period of 3 h; (b) CM with an incubation period of 1 h; (c) CM with an incubation period of 3 h; (d) SM with an incubation period of 1 h; (e) SM with an incubation period of 1 h; and (f) MC with an incubation period of 3 h. EEC=Epididymal epithelial cell, CM=Conditioned medium, SM=Secretome medium, and MC=Medium control.

For tyrosine phosphorylation analysis, molecular analysis was carried out using the Western blot technique (Figure-7). The phosphorylation of this amino acid is an indicator of sperm capacitation. The Western blot results showed that the longer the incubation time, the thicker the band formed using both CM and SM.

## Discussion

### Primary cell culture of mouse caput epididymis and its characteristics

In this study, the isolation and culture of epithelial epididymis caput cells were successfully carried out. The results showed that the best replication time was on day 3, while there was a decrease in cell growth on day 7. This result is similar to those reported in a previous study, where the growth curve increased after the 3^rd^ day and decreased after the 4^th^ day [30]. Mouse epididymis cultures tend to have a relatively short lifespan compared with human epididymis cultures. Studies using the human epithelial epididymis have shown that caput tissue can be cultured for 7–8 passages without any sign of aging [27, 31]. This differs from what was produced in this study, where the mouse epididymal caput tissue began to show aging after the second passage. This difference was due to the lower lifespan of murine cells compared to human cells.

The use of the *Defb20* gene as a marker to confirm the specificity of the caput epididymal cell culture was based on previous research [10]. The results of the research using ddY mice showed that *Defb20* expression was specific only in the caput segment and was not found in other segments of the epididymis or other reproductive organs. ddY mice were used in this study so it was appropriate to use the *Defb20* gene to confirm the specificity of the caput epididymal cells [10]. The analysis of caput cells using the RT-PCR method based on *Defb20* expression showed positive results, so it was concluded that the cultured cells came from the caput of the epididymis. In addition to using *Defb20* expression, RT-PCR analysis was carried out on the *AQP9* gene, which is a marker of principal cells in the epididymis, and positive results were obtained.

### Optimization of incubation time and the response of a medium type of sperm coculture to sperm kinetics and tyrosine phosphorylation

This study revealed a decrease in the percentage of progressive motility of sperm after 2 h of incubation, followed by a significant increase after 3 h of incubation. This substantial increase is possibly due to bonding interactions between compounds in the medium used and the sperm, which lead to improvements in sperm motility and progressivity. The interaction time of ejaculated sperm with epididymal molecules is between 0.5 and 3 h [32]. We identified a negative correlation between VAP value and incubation time.

The results of tyrosine phosphorylation showed a trend of increased activity after 3 h of incubation compared to 1 h of incubation. This agreed with the percentage of sperm motility and progressive motility, but not with the VAP value. The phosphorylation activity can be caused by allosteric modifications, which result in protein conformation changes leading to activation or inactivation. Therefore, the increase and decrease in the tyrosine phosphorylation activity play a role in activating it. These results indicate that increased phosphorylation activity activates a protein that increases the percentage of sperm motility and progressivity.

### Effect of variation of medium for sperm coculture on sperm kinetics

The results showed that sperm coculture on EEC culture, CM, and SM increased the sperm kinetic parameters compared with MC as the control medium. This suggests that epididymis caput cells and/or the molecules they secrete contribute to an increase in sperm kinetic parameters. These results suggest that the mechanism of molecular transfer from the lumen of the epididymis does not require a direct contact between epididymis cells with sperm cells, because this study showed an increase in sperm kinetics parameters after being incubated in CM and SM which both did not contain living epididymal caput cells. The SM group showed lower sperm kinetic values than the EEC culture and CM groups.

Epididymosomes are heterogeneous populations with a size of 50–250 nm [33]. We found that epididymosomes with sizes above 200 nm were absent in the SM group. Epididymosomes contain proteins, lipids, sugars, and non-coding RNA. Some epididymis proteins will join with the epididymosomes, which then bind to sperm [5, 25, 34]. In SM, there are no large epididymosomes, so the interaction of secretory molecules of epididymis cells is not optimal, causing a low response to sperm kinetics.

Sperm cocultured in epididymis cell culture and its derivatives tended to have higher phosphorylation activity compared with the control medium. This result suggests that epididymis cells and secretory factors can increase the tyrosine phosphorylation activity. Post-translational modifications in sperm cells, such as tyrosine phosphorylation, are essential in regulating important processes such as capacitation regulation, motility hyperactivation, and acrosome reactions. Phosphorylation/dephosphorylation is controlled by protein kinases and phosphatases, which cause active or inactive protein functions [35]. A previous study reported that there are six tyrosine-phosphorylated proteins (170, 130, 95, 70, 43, and 25 kDa) in human sperm [36]. Our results showed that the tyrosine residue phosphorylated proteins had molecular weights of 43 kDa, 70 kDa, and 95 kDa, where the highest tyrosine phosphorylation activity was on the tyrosine residue with a molecular weight of 95 kDa. Sperm-oocyte interaction, which allows penetration into the zona pellucida, is one of the roles of the 95 kDa protein.

The results of this study provide insights and information that secretory factors from epididymal caput cells can increase sperm kinetics. In addition, this study shows that CM or SM can be used as a substitute for EEC culture for further analysis and alternative therapy in sperm kinetics in the future. Conditioned medium and SM have the advantage of not being cells, so there is no need to match the donor and recipient to avoid rejection by the immune system. In addition, CM or SM can be produced in large quantities, packaged, and frozen, making the transportation process safer and more accessible. Using a CM is a promising prospect for sperm kinetic therapy.

## Conclusion

The variation medium coculture of CM, SM, and EEC increased sperm kinetics compared to the control medium, with an optimal incubation time of 3 h. For secretome preparation, a 0.25-μm filter is recommended for the isolation of all types of epididymosomes. Conditioned medium and SM may be used as alternatives for the further analysis of secretory factors in the epididymis. This study shows the potency of CM and SM as therapy media for male-assisted reproductive techniques, especially for sperm kinetics abnormalities.

## Authors’ Contributions

LY: Designed and performed the experiments and wrote the manuscript. DAP: Supervision of the study and critical revision of the manuscript for important intellectual content. Both authors have read, reviewed, and approved the final manuscript.
